# Long‐Term Real‐World Survival of Immunotherapy Compared to Chemotherapy for Metastatic Nonsmall Cell Lung Cancer: A Propensity Score‐Matched Analysis

**DOI:** 10.1111/1759-7714.15535

**Published:** 2025-01-13

**Authors:** Kun Kim, Michael Sweeting, Linus Jönsson, Nils Wilking

**Affiliations:** ^1^ Department of Neurobiology, Care Sciences and Society Karolinska Institutet Stockholm Sweden; ^2^ Nordic HTA, AstraZeneca Nordic AB Stockholm Sweden; ^3^ Statistical Innovation, AstraZeneca London UK; ^4^ Department of Oncology‐Pathology Karolinska Institutet Stockholm Sweden

**Keywords:** cancer immunotherapy, crossover effects, metastatic non‐small cell lung cancer, propensity score matching, real‐world evidence, survival analysis

## Abstract

**Background:**

The long‐term real‐world effect of immunotherapy (IO) is uncertain in metastatic nonsmall cell lung cancer (mNSCLC). This retrospective observational study aimed to describe treatment patterns following the introduction of IO, estimate real‐world treatment effects of IO compared to standard of care, and evaluate the impact of introduction of IO on a real‐world population, based on a large dataset of over 10 000 patients with several years of follow‐up.

**Methods:**

Data from routine care of lung cancer patients were extracted from Flatiron Health including those who received either IO or platinum‐based doublet chemotherapy (PBDC) in the first line (1L), or either IO or chemotherapy (CT) in the second line (2L). Real‐world overall survival (rwOS) and real‐world time to next therapy (rwTTNT) were estimated using Cox regression. Flexible parametric models, relaxing proportional hazard assumptions, were used to evaluate long‐term IO effects.

**Results:**

After 1:1 nearest neighbor matching among 16 754 1L and 6548 2L patients, the hazard ratio (HR) was 0.942 (95% CI, 0.902–0.984) in 1L and 0.853 (95% CI, 0.795–0.915) in 2L. Adjusting for crossover effects, HR was 0.887 in 1L and 0.775 in 2L. Over the 7‐year follow‐up, the mean rwOS benefit was 3.2 months for 1L and 2.7 months for 2L. IO significantly delayed rwTTNT in both 1L and 2L. The IO effects increased and persisted over time, with uncertainty in the time‐varying HR estimate.

**Conclusion:**

IO improves survival in patients with mNSCLC, though the effect size is smaller than in trials and long‐term survival estimates are uncertain.

## Graphical abstract

Since 2017, the first‐line (1L) treatment has shifted from PBDC to immunotherapy (IO). PBDC usage has decreased but has not been completely replaced by IO, with similar counts maintained since 2019. Among 16 754 1L patients using 1:1 nearest neighbor matching, the hazard ratio (HR) was 0.942 (95% CI, 0.902–0.984) but it decreased to 0.887 (95% CI, 0.850–0.978) after adjusting for crossover effects.

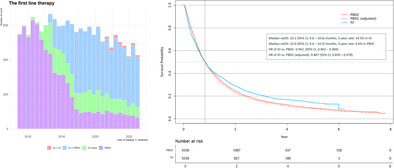

## 1 Introduction

Lung cancer is one of the most common cancers with high mortality [1]. About three in four of the malignancies are diagnosed as nonsmall cell lung cancer (NSCLC). Although about half of these patients have metastatic disease with a 5‐year survival of 5.8% which is significantly lower compared to 68.4% in stage I and 45.1% in stage II in the United States [2], advances in pharmacotherapy have shown promising outcomes for patients with metastatic NSCLC (mNSCLC). Targeted therapies based on molecular testing are considered the first therapeutic options including EGFR mutation, ALK, etc. For those not eligible, international clinical guidelines recommend immunotherapy (IO) mono for PD‐L1 expression ≥ 50% and IO combination with platinum‐based doublet chemotherapy (PBDC) in the first line (1L) and IO mono for PD‐L1 expression ≥ 1% in the second line (2L). Yet, PD‐L1, EGFR mutation, and ALK are the only biomarkers that are acknowledged by high‐level evidence for treatment benefit [3, 4]. IO is thus indicated for 40%–60% of Asian and 70%–90% of Caucasian patients with mNSCLC [5, 6].

Superior overall survival (OS) and progression‐free survival (PFS) have been demonstrated for IO in both patients with squamous and nonsquamous mNSCLC, compared to systematic chemotherapies (CTs) [7, 8, 9, 10, 11, 12, 13, 14]. From recent updates of the pivotal trials, among patients with PD‐L1 expression ≥ 50% in 1L IO mono, 31.9% survived to 5 years (vs. CT: 16.3%) and 12.8% were PFS (vs. CT: not reached) [15]. For 1L patients in IO in combination with PBDC regardless of PD‐L1 expression, 5‐year OS was nearly 20% and 5‐year PFS was 10% [16, 17]. Among patients in 2L IO mono, 5‐year OS and PFS was 14% (vs. docetaxel: 5%) and 9% (vs. docetaxel: 0%), respectively [18, 19]. However, the real‐world treatment effects of IO with longer follow‐up are uncertain.

Outcomes from clinical trials may not be entirely translated into real‐world practice since trial protocols have inclusion and exclusion criteria while real‐world patient populations tend to be more heterogenous including patients with high age, poor performance status (PS), and more comorbidities [2]. High age and the presence of comorbidities may influence the effectiveness of IO in mNSCLC patients. Immunosenescence, the gradual decline of immune function with age, may reduce the ability of elderly patients to mount an effective immune response to IO [20, 21, 22]. Patients with poor PS or multiple comorbidities have been observed with worse survival outcomes with significant toxicities due to the treatment [22]. Treatment patterns in real‐world practice may also diverge from the trials. In clinical trials, patients receive a standardized and carefully monitored treatment regimen, in line with protocols to ensure adherence. In real‐world settings, variations in treatment can occur due to patient preference, physician experience, healthcare system constraints, and treatment adaptation to patient's health status and conditions.

Electronic health record (EHR) data are a suitable data source to include a broad patient population in clinical practice, capturing real‐world treatment patterns, and understanding the real‐world effectiveness of therapies. In recent years, several studies have reported outcomes of IO in mNSCLC in real‐world settings, but most of these studies were single‐arm studies [23, 24, 25, 26, 27, 28, 29, 30, 31, 32, 33] and had a small sample size and a short follow‐up period [34, 35, 36, 37], which makes it difficult to conclude the real‐world treatment effects of IO in a broad mNSCLC patient population (Table 1).

**TABLE 1:** Previous real‐world studies of immunotherapy in advanced nonsmall cell lung cancer.

Study	Country	Data source	Patient population	Diagnosis period	Study drug	Comparator	Median follow‐up	Maximum follow‐up	Study outcomes
Ahn et al. [23]	Korea	Yonsei cancer center	Advanced NSCLC	March 2014–January 2019	Nivolumab or pembrolizumab (*N* = 155)	None	17.0 months	< 60 months	OS, PFS
Faehling et al. [24]	Germany	Expanded access program	Advanced NSCLC	November 2017–October 2018	Durvalumab (*N* = 126)	None	25.1 months	< 30 months	OS, PFS, AE
Murteira et al. [25]	Portugal	National Cancer Registry	Advanced NSCLC (previously treated)	June 2016–October 2018	Pembrolizumab (*N* = 181)	None	9.6 months	< 30 months	OS, PFS, EFS, AE
Okishio et al. [26]	Japan	23 cancer hospitals	Advanced NSCLC	April 2016–December 2016	Nivolumab (*N* = 901)	None	Not reported	< 24 months	OS, PFS, DOR, ORR, DCR
Cortellini et al. [27]	Italy	34 cancer institutions	Metastatic NSCLC, PD‐L1 expression ≥ 50%	January 2017–October 2019	Pembrolizumab (*N* = 1010)	None	14.8 months	< 35 months	ORR, PFS, OS
Grossi et al. [28]	Italy	Expanded access program	Advanced NSCLC (nonsquamous)	June 2015–April 2016	Nivolumab (*N* = 1588)	None	8.1 months	< 30 months	PFS, OS, ORR, DCR
Figueiredo et al. [29]	Portugal	Expanded access program	Advanced NSCLC (previously treated)	June 2015–December 2016	Nivolumab (*N* = 229)	None	17.1 months	< 20 months	PFS, OS
Velcheti et al. [30]	US	Flatiron Health	Metastatic NSCLC (nonsquamous)	May 2017–August 2018	Pembrolizumab plus pemetrexed‐carboplatin (*N* = 283)	None	20.3 months	> 24 months	rwPFS, OS
Crinò et al. [31]	Italy	Expanded access program	Advanced NSCLC (squamous)	April 2015–September 2015	Nivolumab (*N* = 371)	None	7.1 months	< 15 months	OS, ORR, DCR
Lin et al. [32]	Taiwan	National Taiwan University Hospital	Advanced NSCLC	April 2015–August 2017	Nivolumab or pembrolizumab (*N* = 47)	None	12.4 months	< 30 months	PFS, OS
Kobayashi et al. [33]	Japan	Keio Lung Oncology Group	Advanced NSCLC	January–July 2016	Nivolumab (*N* = 142)	None	Not reported	< 12 months	OR, PFS
Dudnik et al. [34]	Israel	4 cancer centers	Advvanced NSCLC, TPS ≥ 50%	June 2016–January 2020	Pembrolizumab monotherapy (*N* = 203) or pembrolizumab + platinum‐based chemotherapy (*N* = 53)	None	22.3 months	> 40 months	OS, TTD
Weis et al. [35]	US	Michigan Medicine	Metastatic or recurrent NSCLC	March 2015–December 2017	Nivolumab (*N* = 81) and atezolizumab (*N* = 43)	None	7.5 months (nivolumab), 4.9 months (atezolizumab)	> 30 months	OS, PFS
Khozin et al. [36]	US	Flatiron Health	Advanced NSCLC	January 2011–December 2017	Nivolumab, pembrolizumab, or atezolizumab (*N* = 5257)	None	> 6 months	> 30 months	rwPFS, rwTTP, rwTTNT, rwTTD
Faehling et al. [37]	Germany	German Cancer Society (DKG)	Advanced NSCLC	January 2006–September 2018	Nivolumab, pembrolizumab, atezolizumab, durvalumab (*N* = 144)	Historic control (*N* = 413)	37.2 months	< 84 months	OS, PFS

Abbreviations: Advanced NSCLC, advanced nonsmall cell lung cancer; AE, adverse events; DCR, disease control rate; DOR, duration of response; EFS, event‐free survival; OR, overall response; ORR, objective response rate; OS, overall survival; PFS, progressive‐free survival; rwPFS, real‐world progression‐free survival; rwTTD, real‐world time to discontinuation; rwTTNT, real‐world time to next therapy; rwTTP, real‐world time to progression; TTD, time‐to‐treatment discontinuation.

We report results from a retrospective observational study to describe treatment patterns following the introduction of IO, estimate real‐world long‐term treatment effects of IO compared to standard of care, and evaluate the impact of introduction of IO on a real‐world patient population in mNSCLC, based on a large‐scale EHR dataset of more than 10 000 patients with several years of follow‐up. This, to our knowledge, is one of the largest cohorts of real‐world IO patients with long follow‐up in the NSCLC literature.

## 2 Material and Methods

### 2.1 Study Design

Data from routine care of lung cancer patients were extracted from the Flatiron Health oncology database, that is, a longitudinal, demographically and geographically diverse database of more than 3 million patient records in the United States [38]. Patients included in this study were aged ≥ 18 years with diagnosis of mNSCLC and had received 1L therapy as either IO, that is, IO mono, IO combined with PBDC (IO + PBDC), or PBDC, or 2L therapy as either IO, that is, IO mono and IO combined with CT (IO + CT), or CT. Patients were excluded if receiving EGFR‐/ALK‐targeted therapies, or clinical study drugs. Patients initially diagnosed with stage I, II, or III cancer, including those who later progressed to stage IV cancer as a recurrence after definitive surgery and chemoradiotherapy, were excluded. Inclusion was restricted further with diagnosis from January 1, 2015, and onward since IO was not authorized for patients with lung cancer before 2015. Patients who did not initiate the treatment within 60 days from metastatic diagnosis or disease progression were excluded.

The primary endpoint was real‐world OS (rwOS) where patients were followed from the date of initiating the treatment until death or patients without date of death were censored at the last confirmed activity date. Details of the method and its validation are described elsewhere [39]. The secondary endpoint was real‐world time to next line of therapy (rwTTNT) where patients were followed from the date of initiating treatment until receipt of the next line of therapy or patients without event were censored at the last confirmed activity date or date of death, whichever occurred first. rwTTNT is an alternative measure to PFS given that data regarding disease progression was not available for all patients. rwOS and rwTTNT were estimated in 1L and 2L, respectively.

### 2.2 Statistical Analysis

Statistical analyses were performed separately for 1L and 2L patients using datasets divided by treatment type and line. The treatment patterns were analyzed using the respective dataset to understand changes in clinical practices following the introduction of IO for mNSCLC.

Given that imbalances in patient characteristics may confound the treatment effect, propensity score analysis was used to minimize the bias. A propensity score was estimated using confounding covariates, that is, age, gender, PS score, smoking status, histology, Charlson Comorbidity Index (CCI), molecular test result (ALK, EGFR, KRAS, and ROS1), PD‐L1 result, PD‐L1 expression level, and history of previous treatment. The year of diagnosis was excluded from the propensity score matching because including it prevented matching the cohorts with a sufficient number of patients. We used a missing category to account for missing values in any of the covariates. IO patients were matched to patients who received PBDC or CT patients using the propensity score, where matching was conducted in 1L and 2L, separately. We attempted 1:1 nearest neighbor matching with a caliper equal to 0.05 of the standard deviation using logistic regression without replacement. This matching approach achieved a good balance and an adequate number of patients for analysis. Other matching approaches were attempted but yielded poor balance or excluded too many patients, as detailed in Table S1. Baseline patient characteristics were described based on the matched cohorts. In addition, we compared treatment effects estimated from matched cohorts with effects estimated using the inverse probability weighting (IPW) method with stabilized weights on the basis of the estimated propensity score [40].

Crossover, where control group patients switch to IO treatment after a period of time, complicates interpretation of treatment effects. The conditions prompting crossover (such as disease progression) may be linked to the primary endpoint, potentially underestimating treatment effects when the estimand of interest is the comparison of IO to a PBDC treatment regimen where IO subsequent therapy is not available. To address this issue the rank‐preserving structural failure time model (RPSFTM) was used to estimate survival outcomes in a situation where crossover could not occur [41, 42, 43, 44]. An acceleration factor, was used to adjust the survival of patients who switched to IO treatments, providing an estimate of the treatment effects in a setting where subsequent IO is not available to patients who received PBDC [45, 46]. We conducted diagnostic tests to ensure the validity of the RPSFTM. Recensoring was performed for control group patients to deal with potential informative censoring. We estimated an HR using a Cox proportional hazards model adjusting for crossover effects using the RPSFTM. In addition, we presented restricted mean survival time (RMST) for rwOS in 1L and 2L, respectively, by setting the restriction time at 7 years.

Finally, we developed a flexible parametric model that uses restricted cubic splines to model time‐varying hazard ratios and used it to visualize long‐term treatment effects [47]. We used a spline function with three internal knots to capture the shape of the log baseline cumulative hazard using the default knot locations at the centiles of the log of the event times. The effect of treatment was allowed to be time‐dependent through a further spline function to model the effect of time since treatment initiation, using three internal knots and default knot locations.

All statistical analyses were conducted in R version 4.2.3.

## 3 Results

**FIGURE 1:**
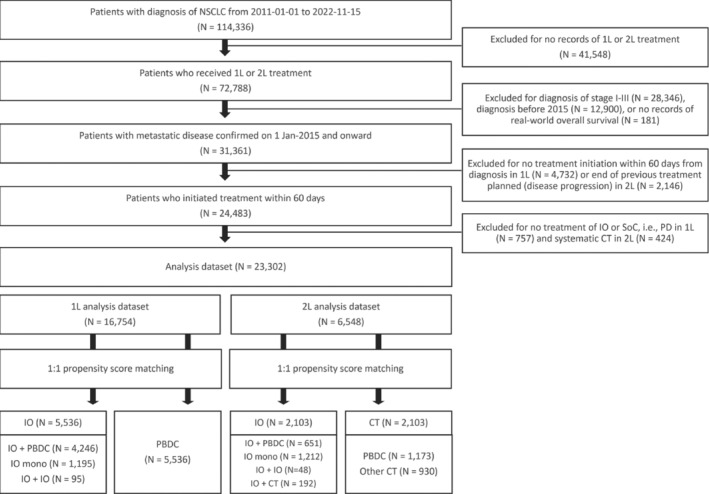
The CONSORT flow diagram. 1:1 nearest neighbor propensity score matching was specified as using a caliper equal to 0.05 of standard deviation and logistic regression without replacement for both 1L and 2L cohorts. CT, chemotherapy; IO, immunotherapy; NSCLC, nonsmall cell lung cancer; PBDC, platinum‐based doublet chemotherapy; SoC, standard of care.

Of 114 336 patients with NSCLC diagnosis in the Flatiron Health database from January 2011 to November 2022, 23 302 met the inclusion criteria having received IO, PBDC, or CT for metastatic disease confirmed after January 1, 2015. In 1L analysis, after 1:1 propensity score matching, 5536 patients who received IO, either IO + PBDC (*N* = 4246), IO mono (*N* = 1195) or IO + IO (*N* = 95), were included in the IO group and 5536 patients were included in the PBDC group. In 2L analysis, after 1:1 propensity score matching, 2103 patients who received an IO, either IO + PBDC (*N* = 651), IO mono (*N* = 1212), IO + IO (*N* = 48), or IO + CT (*N* = 192) were included in the IO group and 2103 patients were included in the CT group (Figure 1).

**FIGURE 2:**
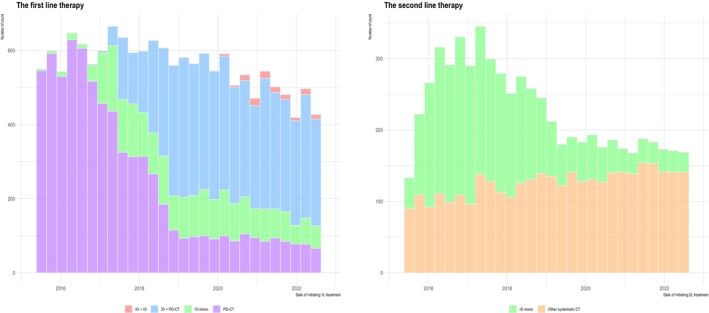
Changes in treatment patterns for metastatic nonsmall cell lung cancer since the introduction of immuno‐oncology, in the first‐ and second‐line therapy. CT, chemotherapy; IO, immuno‐oncology; PBDC, platinum‐based doublet chemotherapy.

Figure 2 illustrates changes in mNSCLC treatment patterns following the introduction of IO. Since 2017, 1L treatment has shifted from PBDC to IO monotherapy and IO + PBDC, while IO + IO (e.g., nivolumab + ipilimumab) has been infrequently used since 2020. PBDC usage has decreased but has not been completely replaced by IO, with similar counts maintained since 2019. In 2L, both IO monotherapy and IO + PBDC have been adopted, yet IO has not fully replaced systemic CT. PBDC and other systemic CT have maintained similar counts over the years, with many patients receiving IO in 2L during the initial years of its introduction, possibly because patients who received 1L IO are less likely to receive 2L IO, explaining the reduced prevalence of 2L IO in later years.

The selected matching method resulted in a well‐balanced matched cohort, with an adequate number of patients included. Diagnostic figures to validate the matching process are presented in Figures S1 and S2. The majority of the covariates had standardized mean differences below 0.1 for both 1L and 2L patients, indicating a successful balance between the treatment and control groups [48, 49].

Baseline patient characteristics before and after matching are described in Table 2. In the 1L matched cohort, the mean age was 68.0 years with the majority of patients male (56.5%), previous smokers (90.0%), and diagnosed with nonsquamous cell carcinoma (71.1%). 24.1% of patients were positive for PD‐L1 test and 9.6% showed high expression of PD‐L1. The maximum follow‐up was 93.5 months, while the median was 8.0 months. In the 2L matched cohort, the mean age was 67.8 years with the majority of patients male (51.3%), previous smokers (85.6%), and diagnosed with nonsquamous cell carcinoma (75.5%). 30.3% of patients were positive for the PD‐L1 test and 15.9% showed high expression of PD‐L1. The maximum follow‐up was 88.1 months, while the median was 7.3 months.

**TABLE 2:** Baseline patient characteristics before and after 1:1 neighbor propensity score matching in 1L and 2L, respectively.

	1L						2L					
	Before matching			After matching			Before matching			After matching		
	PBDC	IO	SMD	PBDC	IO	SMD	CT	IO	SMD	CT	IO	SMD
	*N* = 7809	*N* = 8945		*N* = 5536	*N* = 5536		*N* = 2348	*N* = 4200		*N* = 2103	*N* = 2103	
Age [mean (SD)]	67.29 (9.68)	68.83 (9.8)	0.157	67.91 (9.6)	68.12 (10.13)	0.022	67.21 (10.6)	68.05 (9.9)	0.082	67.71 (10.21)	67.97 (10.18)	0.026
Male, *n* (%)	4404 (56.4)	4908 (54.9)	0.031	3096 (55.9)	3158 (57)	0.023	1209 (51.5)	2268 (54.0)	0.050	1097 (52.2)	1059 (50.4)	0.036
ECOG, *n* (%)			0.199			0.053			0.069			0.020
0	1543 (19.8)	2048 (22.9)		1175 (21.2)	1214 (21.9)		412 (17.5)	762 (18.1)		373 (17.7)	374 (17.8)	
1	2610 (33.4)	3323 (37.1)		2012 (36.3)	1903 (34.4)		886 (37.7)	1540 (36.7)		790 (37.6)	773 (36.8)	
2	1081 (13.8)	1358 (15.2)		816 (14.7)	853 (15.4)		365 (15.5)	747 (17.8)		348 (16.5)	356 (16.9)	
3	238 (3.0)	300 (3.4)		167 (3.0)	202 (3.6)		92 (3.9)	163 (3.9)		81 (3.9)	83 (3.9)	
4	13 (0.2)	20 (0.2)		10 (0.2)	9 (0.2)		7 (0.3)	11 (0.3)		7 (0.3)	6 (0.3)	
Missing	2324 (29.8)	1896 (21.2)		1356 (24.5)	1355 (24.5)		586 (25.0)	977 (23.3)		504 (24)	511 (24.3)	
Smoking history, *n* (%)			0.054			0.026			0.15			0.012
Yes	7035 (90.1)	8102 (90.6)		4991 (90.2)	4970 (89.8)		1948 (83.0)	3704 (88.2)		1795 (85.4)	1804 (85.8)	
No	748 (9.6)	835 (9.3)		542 (9.8)	559 (10.1)		396 (16.9)	489 (11.6)		304 (14.5)	295 (14)	
Unknown	26 (0.3)	8 (0.1)		3 (0.1)	7 (0.1)		4 (0.2)	7 (0.2)		4 (0.2)	4 (0.2)	
Diagnose year, *n* (%)			1.738			1.533			0.187			0.285
2015	2153 (27.6)	21 (0.2)		1237 (22.3)	17 (0.3)		516 (22.0)	689 (16.4)		500 (23.8)	304 (14.5)	
2016	2174 (27.8)	156 (1.7)		1450 (26.2)	110 (2.0)		394 (16.8)	868 (20.7)		375 (17.8)	352 (16.7)	
2017	1414 (18.1)	1005 (11.2)		1081 (19.5)	553 (10.0)		404 (17.2)	777 (18.5)		377 (17.9)	358 (17)	
2018	779 (10.0)	1573 (17.6)		638 (11.5)	969 (17.5)		288 (12.3)	621 (14.8)		244 (11.6)	314 (14.9)	
2019	368 (4.7)	1803 (20.2)		309 (5.6)	1180 (21.3)		233 (9.9)	434 (10.3)		188 (8.9)	282 (13.4)	
2020	370 (4.7)	1668 (18.6)		322 (5.8)	991 (17.9)		211 (9.0)	366 (8.7)		172 (8.2)	220 (10.5)	
2021	335 (4.3)	1559 (17.4)		307 (5.5)	988 (17.8)		219 (9.3)	327 (7.8)		181 (8.6)	199 (9.5)	
2022	216 (2.8)	1160 (13.0)		192 (3.5)	728 (13.2)		83 (3.5)	118 (2.8)		66 (3.1)	74 (3.5)	
Histology			0.112			0.008			0.237			0.014
Nonsquamous cell	5435 (69.6)	6671 (74.6)		3946 (71.3)	3929 (71)		1815 (77.3)	2815 (67.0)		1587 (75.5)	1590 (75.6)	
Squamous cell	1954 (25.0)	1850 (20.7)		1307 (23.6)	1317 (23.8)		433 (18.4)	1177 (28.0)		422 (20.1)	414 (19.7)	
Unknown	420 (5.4)	424 (4.7)		283 (5.1)	290 (5.2)		100 (4.3)	208 (5.0)		94 (4.5)	99 (4.7)	
CCI, *n* (%)			0.129			0.037			0.037			0.001
≥ 7	2305 (29.5)	3157 (35.3)		1830 (33.1)	1925 (34.8)		829 (35.3)	1409 (33.5)		731 (34.8)	732 (34.8)	
< 7	5429 (69.5)	5678 (63.5)		3641 (65.8)	3544 (64.0)		1519 (64.7)	2791 (66.5)		1372 (65.2)	1371 (35.2)	
Missing	75 (1.0)	110 (1.2)		65 (1.2)	67 (1.2)		—	—				
ALK status, *n* (%)			0.273			0.010			0.241			0.020
Negative	5376 (68.8)	7210 (80.6)		4160 (75.1)	4140 (74.8)		1803 (76.8)	3204 (76.3)		1651 (78.5)	1651 (78.5)	
Missing	2364 (30.3)	1678 (18.8)		1334 (24.1)	1351 (24.4)		456 (19.4)	975 (23.2)		435 (20.7)	431 (20.5)	
Positive	69 (0.9)	57 (0.6)		42 (0.8)	45 (0.8)		89 (3.8)	21 (0.5)		17 (0.8)	21 (1.0)	
EGFR status, *n* (%)			0.227			0.010			0.115			0.018
Negative	5361 (68.7)	7012 (78.4)		4066 (73.4)	4048 (73.1)		1712 (72.9)	3031 (72.2)		1505 (71.6)	1522 (72.4)	
Missing	2089 (26.8)	1585 (17.7)		1219 (22)	1241 (22.4)		393 (16.7)	842 (20.0)		367 (17.5)	356 (16.9)	
Positive	359 (4.6)	348 (3.9)		251 (4.5)	247 (4.5)		243 (10.3)	327 (7.8)		231 (11)	225 (10.7)	
KRAS status, *n* (%)			0.464			0.027			0.048			0.025
Negative	2485 (31.8)	3931 (43.9)		2322 (41.9)	2252 (40.7)		995 (42.4)	1738 (41.4)		867 (41.2)	871 (41.4)	
Missing	4236 (54.2)	2876 (32.2)		2184 (39.5)	2213 (40.0)		924 (39.4)	1746 (41.6)		851 (40.5)	830 (39.5)	
Positive	1088 (13.9)	2138 (23.9)		1030 (18.6)	1071 (19.3)		429 (18.3)	716 (17.0)		385 (18.3)	402 (19.1)	
ROS1 status, *n* (%)			0.477			0.038			0.142			0.005
Negative	4314 (55.2)	6898 (77.1)		3918 (70.8)	3827 (69.1)		1596 (68.0)	2816 (67.0)		1424 (67.7)	1429 (68.0)	
Missing	3468 (44.4)	2015 (22.5)		1593 (28.8)	1687 (30.5)		707 (30.1)	1365 (32.5)		660 (31.4)	655 (31.1)	
Positive	27 (0.3)	32 (0.4)		25 (0.5)	22 (0.4)		45 (1.9)	19 (0.5)		19 (0.9)	19 (0.9)	
PD‐L1 status, *n* (%)			0.561			0.066			0.065			0.005
Negative	1595 (20.4)	1632 (18.2)		1281 (23.1)	1203 (21.7)		480 (20.4)	800 (19.0)		419 (19.9)	415 (19.7)	
Missing	4901 (62.8)	3672 (41.1)		2997 (54.1)	2923 (52.8)		1195 (50.9)	2074 (49.4)		1049 (49.9)	1049 (49.9)	
Positive	1313 (16.8)	3641 (40.7)		1258 (22.7)	1410 (25.5)		673 (28.7)	1326 (31.6)		635 (30.2)	639 (30.4)	
PD‐L1 expression, *n* (%)			0.52			0.068			0.05			0.020
Missing	6977 (89.3)	6315 (70.6)		4736 (85.5)	4622 (83.5)		1876 (79.9)	3309 (78.8)		1663 (79.1)	1659 (78.9)	
High	477 (6.1)	2114 (23.6)		477 (8.6)	587 (10.6)		359 (15.3)	642 (15.3)		337 (16)	332 (15.8)	
Low	355 (4.5)	516 (5.8)		323 (5.8)	327 (5.9)		113 (4.8)	249 (5.9)		103 (4.9)	112 (5.3)	
1L treatment, *n* (%)									0.643			0.039
IO							846 (36.0)	733 (17.5)		712 (33.9)	683 (32.5)	
PBDC							866 (36.9)	2830 (67.4)		529 (25.2)	518 (24.6)	
Other							636 (27.1)	637 (15.2)		862 (41.0)	902 (42.9)	

Abbreviations: CCI, Charlson comorbidity index; CT, chemotherapy; ECOG, Eastern Cooperative Oncology Group performance status scale; IO, immunotherapy; PBDC, platinum‐based doublet chemotherapy.

**FIGURE 3:**
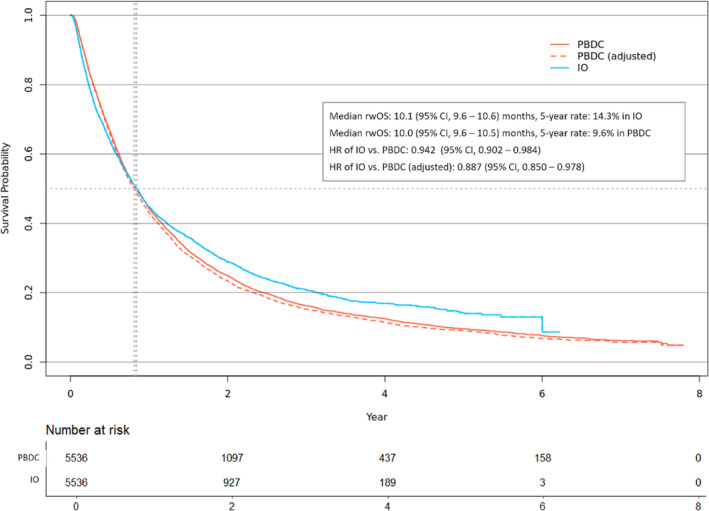
Real‐world overall survival in propensity score‐matched cohorts in the first‐line therapy. IO, immuno‐oncology; PBDC, platinum‐based doublet chemotherapy; rwOS, real‐world overall survival.

**FIGURE 4:**
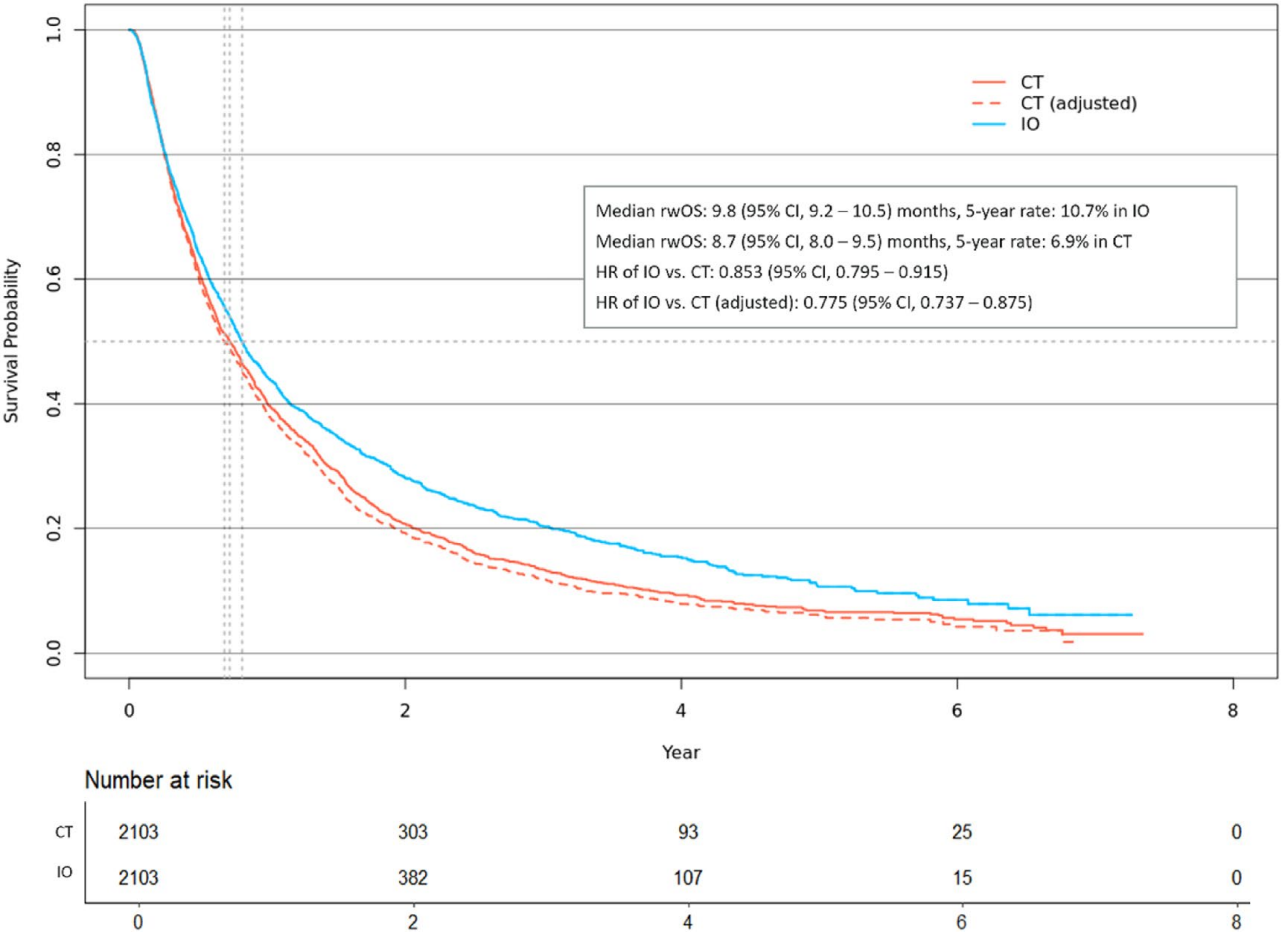
Real‐world overall survival in propensity score‐matched cohorts in the second‐line therapy. CT, chemotherapy; IO, immuno‐oncology; rwOS, real‐world overall survival.

**FIGURE 5:**
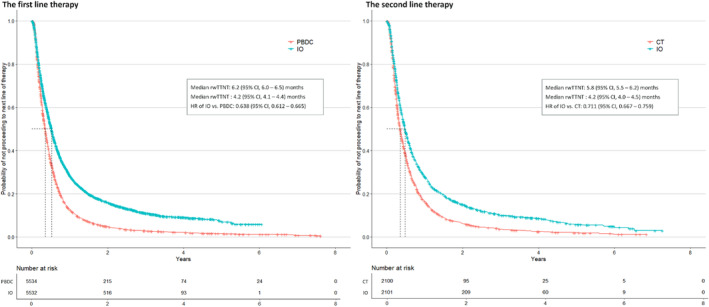
Real‐world time to next line of therapy in propensity score‐matched cohorts in the first‐ and second‐line therapy. CT, chemotherapy; IO, immunotherapy; NSCLC, nonsmall cell lung cancer; PBDC, platinum‐based doublet chemotherapy; rwTTNT, real‐world time to next therapy.

Figures 3 and 4 illustrate rwOS for 1L and 2L patients, respectively. In 1L, IO patients demonstrated a slight survival benefit over PBDC patients, with an HR for all‐cause mortality of 0.942 (95% CI, 0.902–0.984). Adjusting for crossover effects in the PBDC arm increased the benefit, yielding an HR of 0. 0.887 (95% CI, 0.850–0.978). In 2L, IO patients also showed a survival advantage over CT patients, with an HR of 0.853 (95% CI, 0.795–0.915), which improved to 0.775 (95% CI, 0.737–0.875) after crossover adjustment. Over the 7‐year follow‐up period, RMST for rwOS was 8.5 months (SE, 2.8) for 1L IO and 5.4 months (SE, 0.6) for 1L PBDC, while for 2L RMST, the estimate was 6.5 months (SE, 1.2) for 2L IO and 3.8 months (SE, 0.7) for 2L CT. These results align with those from the IPW method, although the HR was higher in 2L using IPW due to the larger number of CT patients included (Figure S3). The benefit of IO appeared larger in rwTTNT for both 1L and 2L, as shown in Figure 5. IO significantly delayed the initiation of the next line of therapies compared to PBDC in 1L (HR = 0.638, 95% CI, 0.612–0.665) and CT in 2L (HR = 0.711, 95% CI, 0.667–0.759).

**FIGURE 6:**
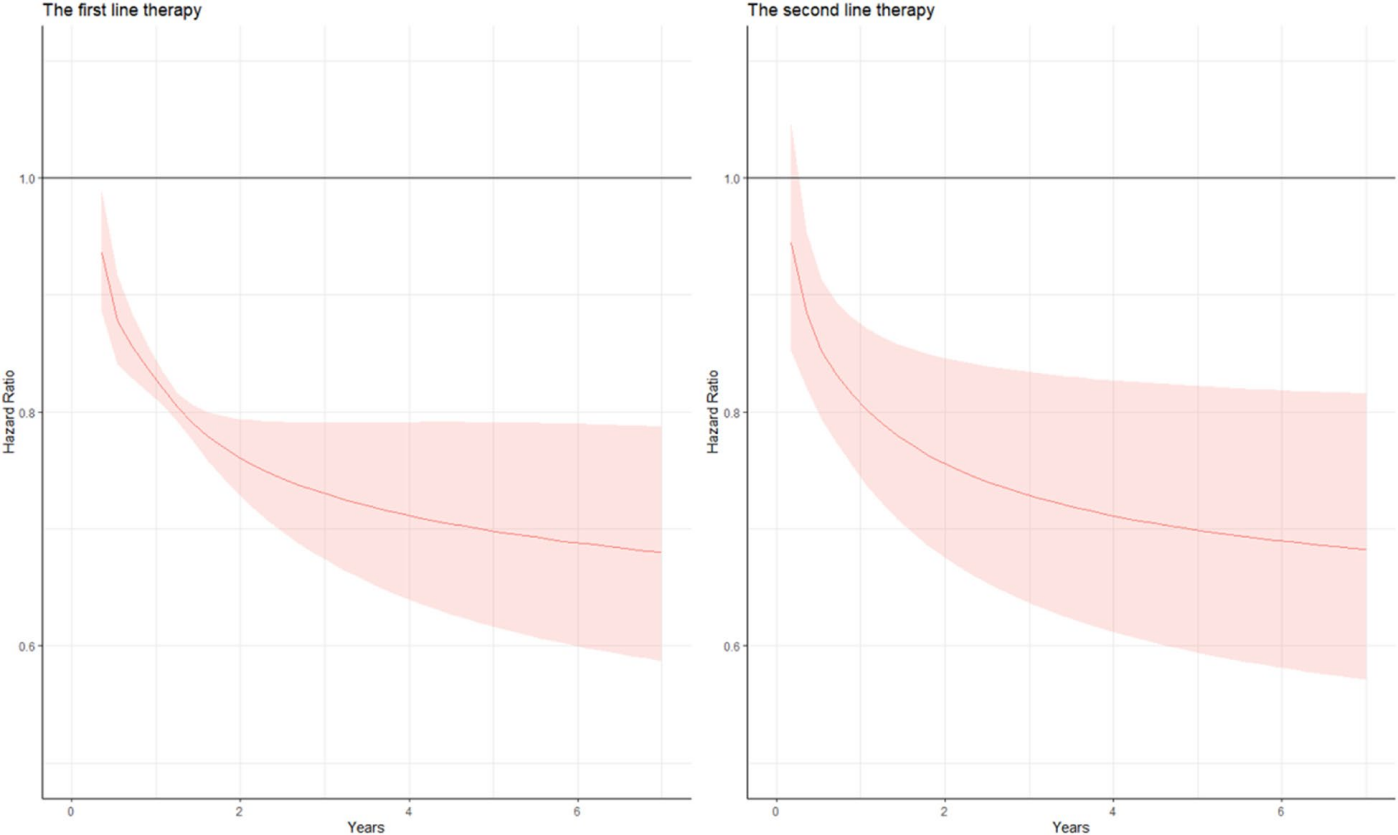
Time‐variant hazard ratios over time of immunotherapy versus platinum‐doublet chemotherapy using restricted cubic spline models in the first‐ and second‐line therapy.

The Schoenfeld residual plots (Figure S4) suggest that the proportional hazards assumption is not met. Figure 6 further supports nonproportional hazards, as the flexible parametric model estimates an HR that decreased rapidly from the null over the first year indicating a delayed treatment effect, which is also visible in the survival curves (Figure S5). Between years 1 and 6, the HR continued to decrease suggesting a continued significant effect of IO, though there was considerable uncertainty in the estimate after 2 years.

## 4 Discussion

Randomized controlled trials are the gold standard for studying causal effects of treatments, yet trials tend to include younger and healthier patients, which limits generalizability to translate the outcomes into real‐world practice. To our knowledge, this is the first real‐world study to present the long‐term clinical outcomes of IO in a large number of mNSCLC patients using propensity score matching methods to minimize the disparity between treated and control patients, which may lead to a biased estimate of the relative treatment effects. We present novel insights into the evolving treatment patterns for mNSCLC, that while IO has become the dominant therapy since its introduction, PBDC and other systemic CT have continued to remain in clinical practice. This analysis of treatment shifts over several years, capturing the expansion of IO as the preferred treatment option in real‐world practice. We found that IO patients with mNSCLC in both 1L and 2L compared to PBDC (1L) and CT (2L) continued to benefit for several years, prolonging OS and delaying the next line of therapies. After accounting for the crossover effects the relative treatment effects of IO were slightly stronger.

There are few aspects of our findings that need to be interpreted with caution. In the 1L matched cohort, the rwOS curves crossed during the initial year. This crossing may be attributed to patient selection, immune‐related adverse events or delayed treatment effect associated with IO, as previously reported in the literature [50, 51, 52]. Our estimation for median rwOS was shorter than that of the trials where the patients, who had life expectancy of at least 3 months, PS ≤ 1, adequate organ function, no history of prior malignancy, no active autoimmune disease, no chronic use of chronic systemic steroids, no active metastases in central nervous system, no history of or active fatal infectious disease, were included [15, 16, 17, 18, 19]. Patients with brain metastases were included in these trials, but only if their disease was stable, as per the inclusion and exclusion criteria. In our analysis, a significant proportion of the patients were aged 70 years or older (46.5%), had a PS score of 2 or higher (18.6%), and had a CCI score of 7 or higher (33.9%) in the 1L matched cohort, and aged 70 years or older (46.3%), had a PS score of 2 or higher (20.9%), and had a CCI score of 7 or higher (34.8%) in the 2L matched cohort. Among the patients in the analysis dataset, 29.1% had bone metastasis, 16.2% had brain metastasis, and many had other chronic conditions such as essential hypertension (27.3%), long‐term drug therapy (20.2%), and type II diabetes, with or without complications (9.6%). When the patients who were older and had PS ≥ 2 and CCI ≥ 7 were excluded to emulate the trial population, the HR was lower without crossover adjustment, being 0.841 (95% CI, 0.772–0.916) in 1L and 0.810 (95% CI, 0.741–0.899) in 2L.

The RPSFTM assumes a common treatment effect of IO across time and treatment line when removing the crossover effects of subsequent IO from the control group. Although diagnostic tests confirmed the feasibility of applying the RPSFTM, the results should be interpreted carefully. We calculated standard errors and confidence intervals using bootstrapping and presented the crossover‐adjusted outcomes with recensoring to address potential informative censoring in the control group. Without recensoring, the treatment effect was estimated to be slightly lower, with an HR of 0.912 (95% CI, 0.849–0.975) in 1L and 0.820 (95% CI, 0.748–0.900) in 2L.

We developed flexible parametric survival models that allowed a time‐varying effect of treatment to attain the best fit to the analysis data (Figure S5). The HR estimated by the flexible parametric model drops sharply in the first year and then goes down more smoothly. The flexible parametric model estimates considerable uncertainty in the HR estimate after year 2, although the HR remained highly statistically significant.

Our study has several limitations. First, this study is retrospective in nature and was not designed to establish causality between IO and clinical outcomes. Instead, our findings represent an association between the use of IO and improvements in rwOS and rwTTNT in patients with mNSCLC. While propensity score matching minimized confounding, it only addressed observed variables. Any unobserved factors could still have influenced the outcomes, and we were unable to control for these potential confounders. Second, our findings are based on a retrospective cohort analysis using electronic medical records, with inherent variability in accuracy and data availability. The number of metastases and their locations were not available, which might be associated with poor prognosis. Information to confirm progressive disease, adherence to treatment, PS score, and biomarker tests were not available for all patients. The CCI score was derived by linking to the diagnosis table, which is also an incomplete data source, primarily used by practices for billing and insurance purposes. Our findings are restricted due to these missed and incomplete prognostic factors that propensity score matching was not able to control although a missing category was added to account for missing values. Third, the Flatiron Health oncology database has limitations when interpreting mortality data. Although a validation exercise reported 91% sensitivity for mortality data among mNSCLC patients, this accuracy may vary for newly collected data. The National Death Index (NDI), the most comprehensive data source, has a lag time of up to 22 months, which may impact the accuracy of mortality data interpretation [53]. Fourth, the included patients do not represent the entire real‐world population of mNSCLC patients because of inclusion and exclusion criteria within this study, which led to the exclusion of patients who did not adhere to standard treatment regimens or clinical guidelines. Although this study includes a broader population than the previous clinical trials, the survival outcomes may not fully represent all real‐world IO patients. Finally, TTNT might be measured systematically shorter than PFS due to various reasons such as an adherence issue, adverse events and any unknown reason for discontinuation rather than disease progression [54].

## 5 Conclusions

IO improves OS and delay receiving next line of therapies for patients with mNSCLC in routine care use. The mean rwOS benefit of IO compared to the previous standard of care was 3.2 months for 1L and 2.7 months for 2L over the 7‐year follow‐up period. However, the effect size is smaller than that observed in clinical trials and the long‐term survival estimates were considerably uncertain.

## Author Contributions

**Kun Kim:** conceptualization (equal), orignal draft (lead), formal analysis (lead), review and editing (equal). **Michael Sweeting:** conceptualization (equal), formal analysis (equal), review and editing (equal). **Linus Jönsson:** conceptualization (equal), formal analysis (equal), review and editing (equal). **Nils Wilking:** conceptualization (lead), review and editing (equal).

## Conflicts of Interest

The authors declare no conflicts of interest.

## Supporting information


**Figure S1.** Distributional balance of the propensity score and absolute standardized mean difference on covariates before and after 1:1 nearest neighbor matching in the first‐line therapy.
**Figure S2.** Distributional balance of the propensity score and absolute standardized mean difference on covariates before and after 1:1 nearest neighbor matching in the second‐line therapy.
**Figure S3.** Real‐world overall survival estimated by propensity score using the inverse probability weighting method with stabilized weights in the first‐ and second‐line therapy.
**Figure S4.** Schoenfeld residuals plots for real‐world overall survival in the first‐ and second‐line therapy.
**Figure S5.** Flexible parametric (spline using three knots) models and fitness to the real‐world overall survival in the first‐ and second‐line therapy.

**Table S1.** Summary of diagnostic statistics based on propensity score matching methods attempted for 1L and 2L cohorts.

## Data Availability

The data utilized in this study were sourced from the Flatiron Health oncology database, accessible for commercial purposes via a license agreement. Due to licensing restrictions, the data cannot be publicly shared. Researchers interested in conducting similar analyses are encouraged to reach out to Flatiron Health.
