# Correction to “Long‐Term Real‐World Survival of Immuno‐ therapy Compared to Chemotherapy for Metastatic Nonsmall Cell Lung Cancer: A Propensity Score‐Matched Analysis”

**DOI:** 10.1111/1759-7714.70038

**Published:** 2025-03-24

K. Kim, M. Sweeting, L. Jönsson, and N. Wilking, “Long‐Term Real‐World Survival of Immunotherapy Compared to Chemotherapy for Metastatic Nonsmall Cell Lung Cancer: A Propensity Score‐Matched Analysis,” *Thoracic Cancer* 16, no. 1 (2025): e15535.10.1111/1759-7714.15535PMC1172985239806827

Figure 2 has been corrected to include five data labels for “The second line therapy”.

Below is the correct Figure 2.

**FIGURE 2:**
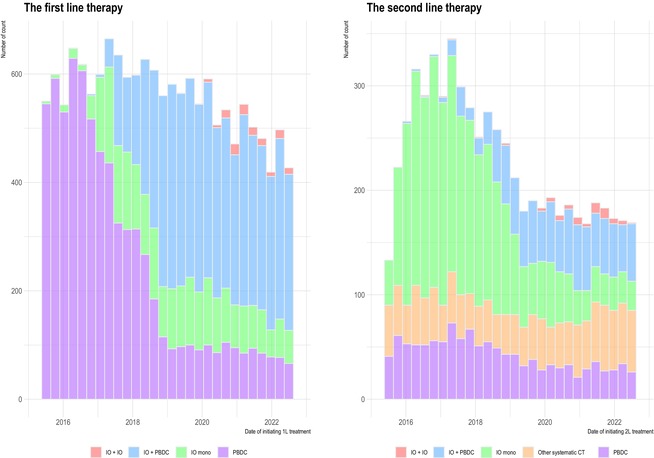
Changes in treatment patterns for metastatic nonsmall cell lung cancer since the introduction of immuno‐oncology, in the first‐ and second‐line therapy. CT, chemotherapy; IO, immuno‐oncology; PBDC, platinum‐based doublet chemotherapy.

