# Supplementary material for: Long‐Term Real‐World Survival of Immunotherapy Compared to Chemotherapy for Metastatic Nonsmall Cell Lung Cancer: A Propensity Score‐Matched Analysis
Source: Thorac Cancer. 2025 Jan 13;16(1):e15535. doi: 10.1111/1759-7714.15535 (PMC11729852; doi:10.1111/1759-7714.15535)
Supplement: Supplementary file 1 — Figure S1. Distributional balance of the propensity score and absolute standardized mean difference on covariates before and after 1:1 nearest neighbor matching in the first‐line therapy. Figure S2. Distributional balance of the propensity score and absolute standardized mean difference on covariates before and after 1:1 nearest neighbor matching in the second‐line therapy. Figure S3. Real‐world overall survival estimated by propensity score using the inverse probability weighting method with stabilized weights in the first‐ and second‐line therapy. Figure S4. Schoenfeld residuals plots for real‐world overall survival in the first‐ and second‐line therapy. Figure S5. Flexible parametric (spline using three knots) models and fitness to the real‐world overall survival in the first‐ and second‐line therapy. [file TCA-16-e15535-s001.docx]

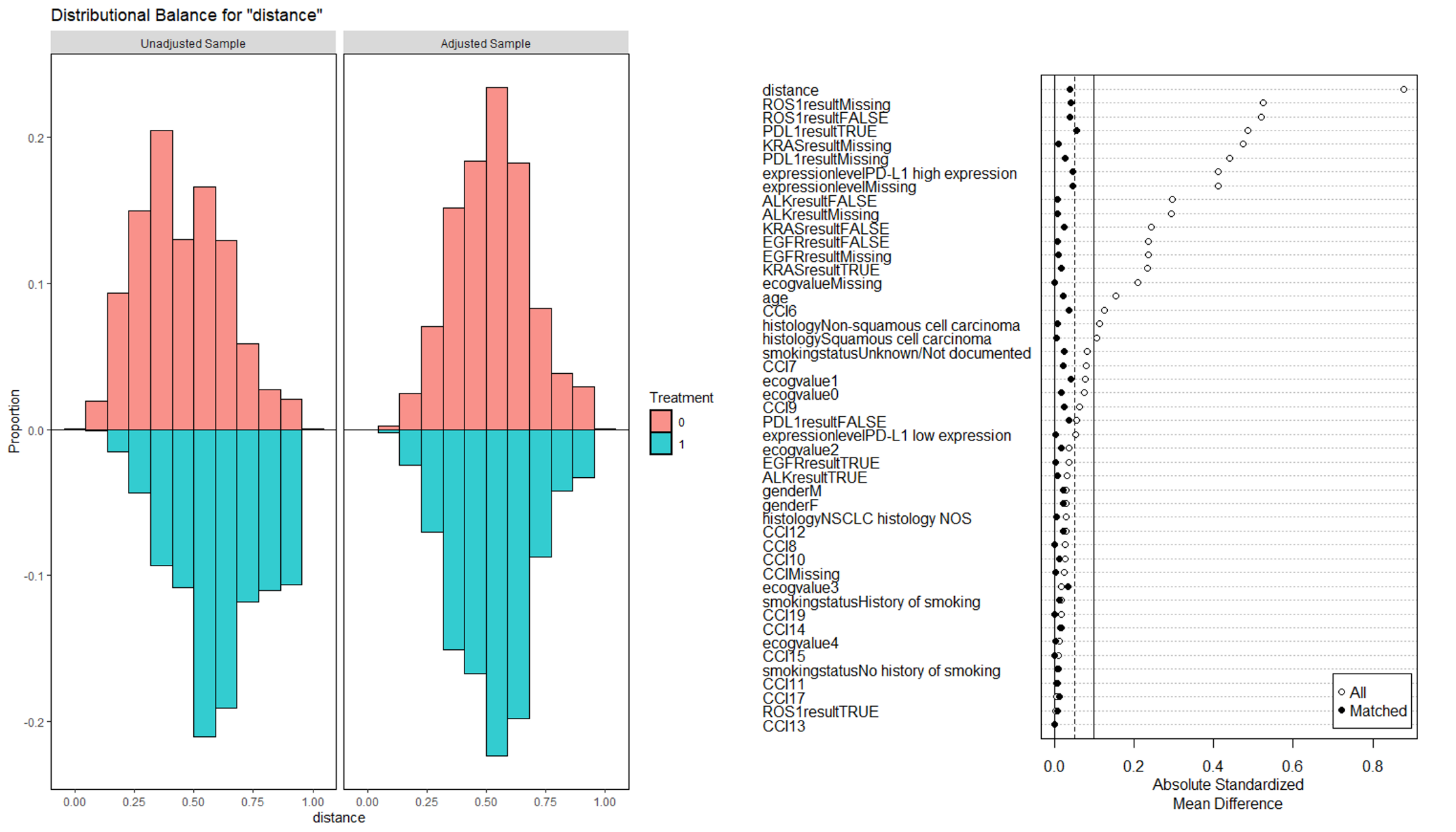
Supplemental figure 1. Distributional balance of the propensity score and absolute standardized mean difference on covariates before and after 1:1 nearest neighbor matching in the first line therapy


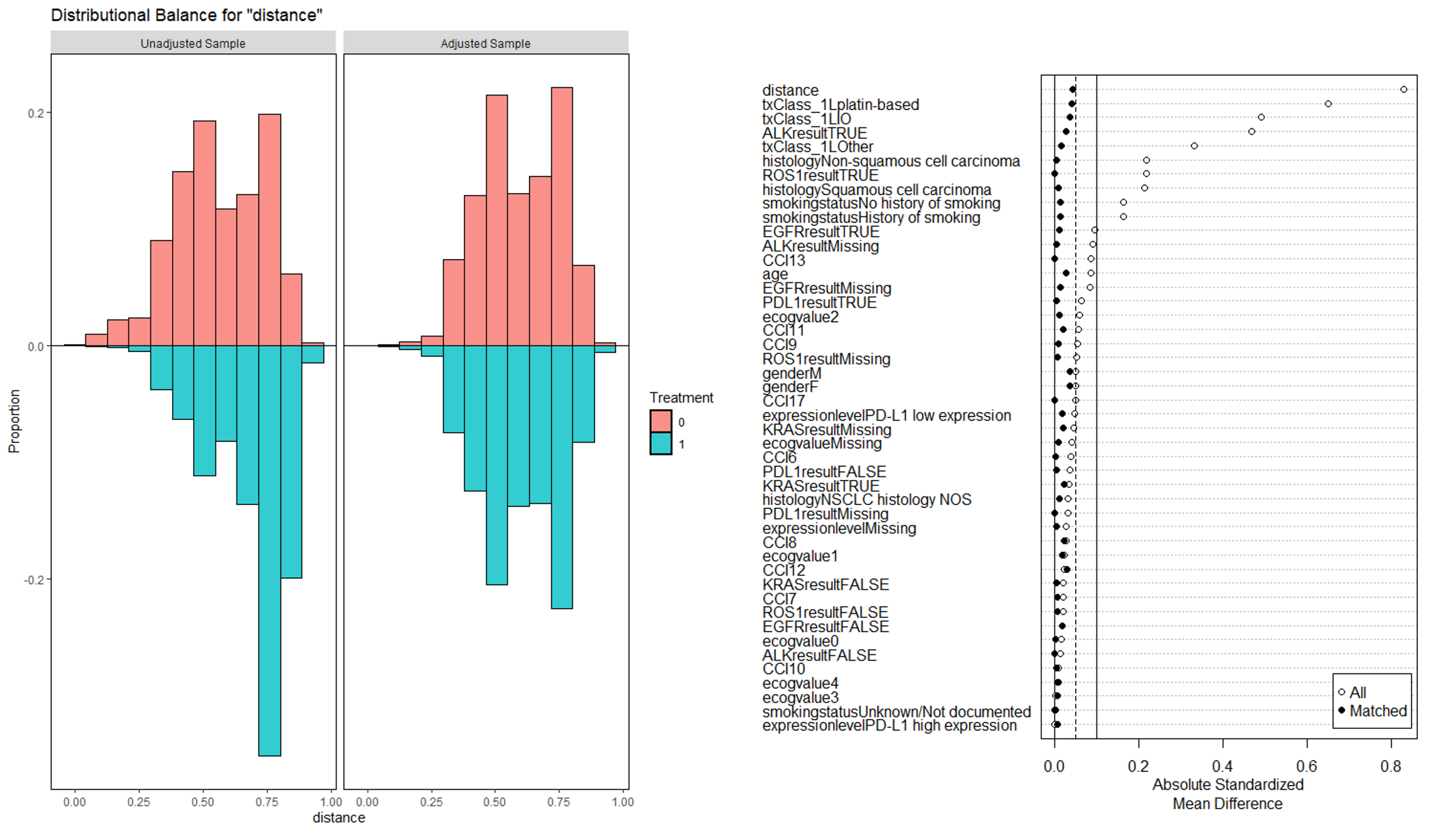


## Supplemental figure 2. Distributional balance of the propensity score and absolute standardized mean difference on covariates before and after 1:1 nearest neighbor matching in the second line therapy


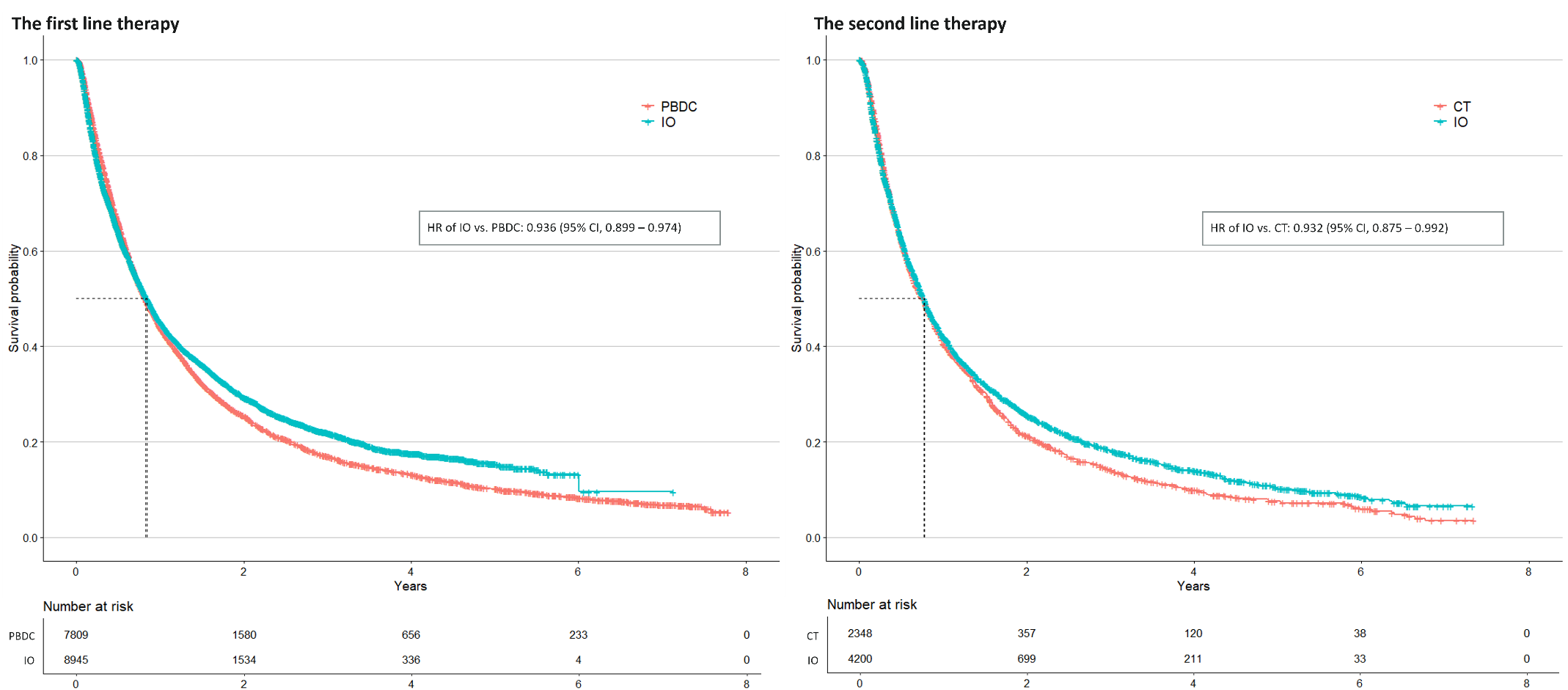


## Supplemental figure 3. Real-world overall survival estimated by propensity score using the inverse probability weighting method with stabilized weights in the first- and second-line therapy

*CT: Chemotherapy, IO: Immuno-oncology, PBDC: Platinum-based doublet chemotherapy


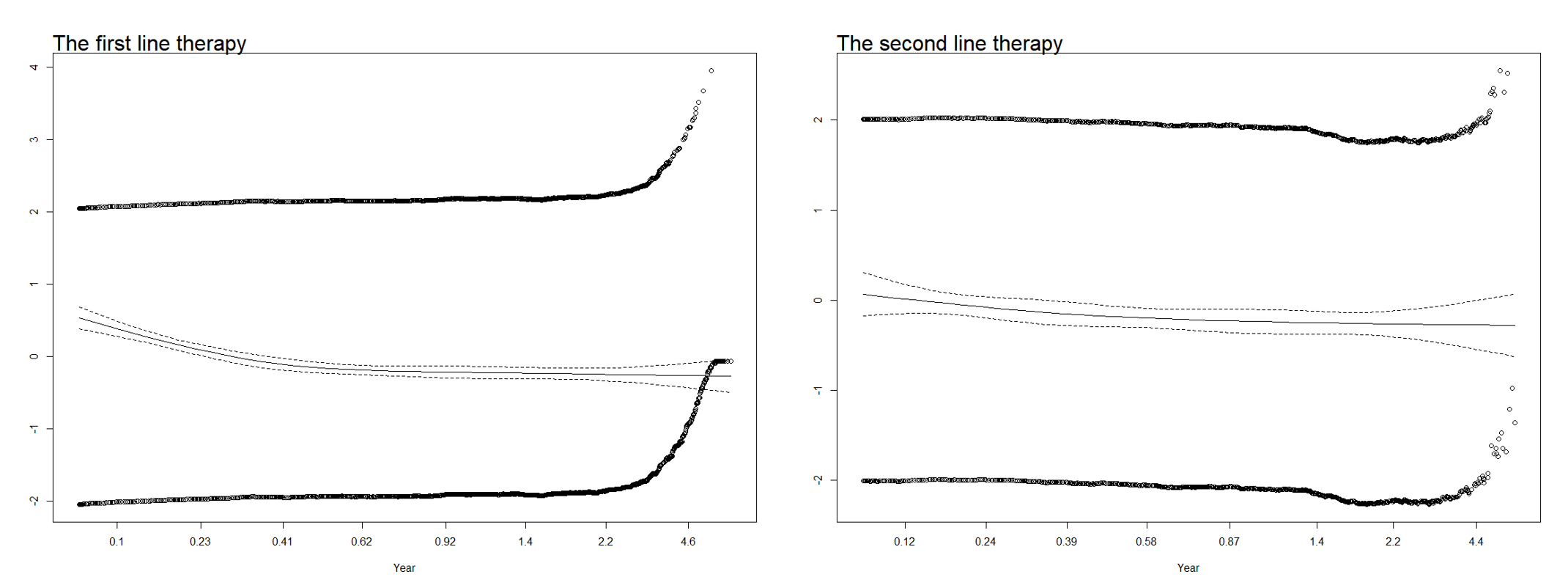


## Supplemental figure 4. Schoenfeld residuals plots for real-world overall survival in the first- and second-line therapy


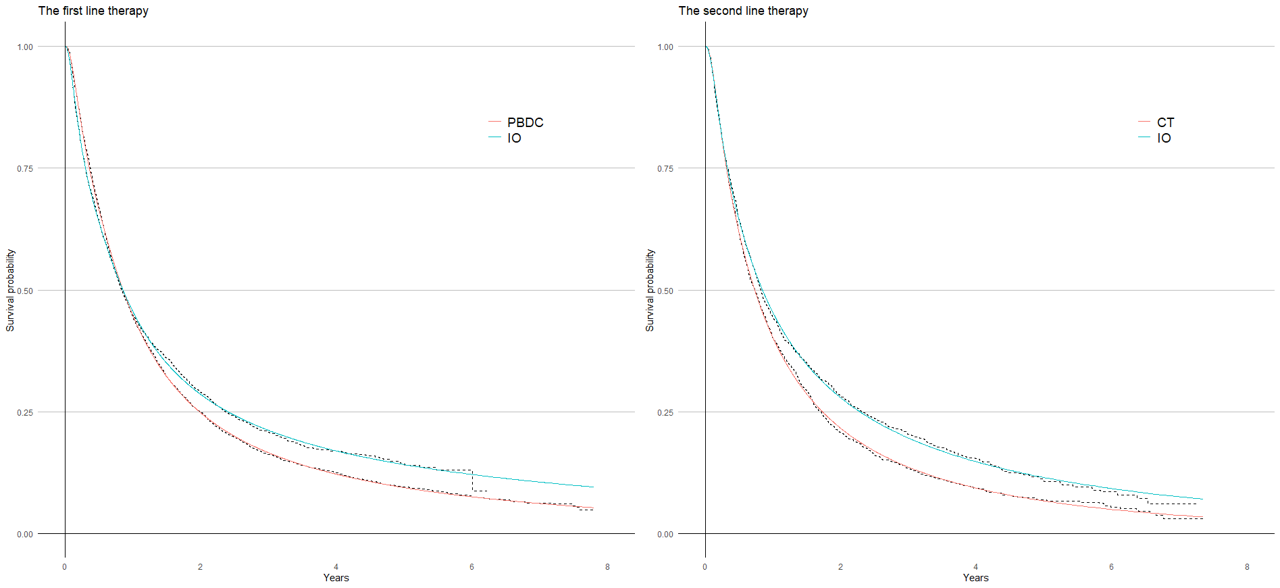


## Supplemental figure 5. Flexible parametric (spline using three knots) models and fitness to the real-world overall survival in the first- and second-line therapy

*CT: Chemotherapy, IO: Immuno-oncology, PBDC: Platinum-based doublet chemotherapy
