# Supplementary material for: Long‐Term Real‐World Survival of Immunotherapy Compared to Chemotherapy for Metastatic Nonsmall Cell Lung Cancer: A Propensity Score‐Matched Analysis
Source: Thorac Cancer. 2025 Jan 13;16(1):e15535. doi: 10.1111/1759-7714.15535 (PMC11729852; doi:10.1111/1759-7714.15535)
Supplement: Supplementary file 2 — Table S1. Summary of diagnostic statistics based on propensity score matching methods attempted for 1L and 2L cohorts. [file TCA-16-e15535-s002.docx]

## Supplemental table 1. Summary of diagnostic statistics based on propensity score matching methods attempted for 1L and 2L cohorts

| Method | Control (n=) | Treatment (n=) | Standardized mean difference | Variance ratio | Number of unbalanced variables (>0.05) |
| --- | --- | --- | --- | --- | --- |
| 1L |  |  |  |  |  |
| Before matching | 7809 | 8945 | 2.0759 | 0.5954 | 20 of 45 |
| 1:1 nearest neighbor, caliper =0.05, ratio = 1, replace = F | 5536 | 5536 | 0.0382 | 1.0424 | 1 of 45 |
| Quick, ratio = 1, replace = F | 2340.94 | 8945 | 0.0000 | 0.9994 | 6 of 45 |
| Subclass, ratio = 1, replace = F | 7809 | 8945 | 0.0526 | 0.9091 | 3 of 45 |
| 1:1 nearest neighbor, caliper =0.05, ratio = 3, replace = F | 6386.18 | 5536 | 0.0419 | 1.0332 | 1 of 45 |
| Exact, ratio = 1, replace = F | 1283.26 | 2003 | 0 | 0.9997 | 0 of 45 |
| Coarsened exact, ratio = 1 | 1958.08 | 3934 | 0.0078 | 1.004 | 0 of 45 |
| 1:1 nearest neighbor, caliper = 0.05, ratio = 1, replace = T, reuse.max=3 | 3379.99 | 8241 | 0.0106 | 1.0313 | 6 of 45 |
| 1:1 nearest neighbor, caliper = 0.05, ratio = 1, replace = T, reuse.max=5 | 2659.09 | 8882 | 0.0077 | 1.0222 | 5 of 45 |
| 1:1 nearest neighbor, caliper = 0.05, ratio = 3, replace = T, reuse.max=3 | 4730.38 | 8241 | 0.0196 | 1.0322 | 3 of 45 |
| 2L |  |  |  |  |  |
| Before matching | 2348 | 4200 | 0.8691 | 0.7530 | 20 of 44 |
| 1:1 nearest neighbor, caliper =0.05, ratio = 1, replace = F | 2103 | 2103 | 0.0427 | 1.0308 | 0 of 44 |
| Quick, ratio = 1, replace = F | 842.33 | 4200 | 0.0003 | 0.9990 | 1 of 44 |
| Subclass, ratio = 1, replace = F | 2348 | 4200 | 0.0511 | 0.8701 | 1 of 44 |
| 1:1 nearest neighbor, caliper =0.05, ratio = 3, replace = F | 2191.58 | 2103 | 0.0442 | 1.0267 | 0 of 44 |
| Exact, ratio = 1, replace = F | 133.13 | 178 | 0 | 0.9981 | 0 of 44 |
| Coarsened exact, ratio = 1 | 327.22 | 713 | 0.0036 | 0.9715 | 0 of 44 |
| 1:1 nearest neighbor, caliper = 0.05, ratio = 1, replace = T, reuse.max=3 | 1396.73 | 3688 | 0.0233 | 1.0394 | 0 of 44 |
| 1:1 nearest neighbor, caliper = 0.05, ratio = 1, replace = T, reuse.max=5 | 1093.35 | 4064 | 0.0066 | 1.0141 | 0 of 44 |
| 1:1 nearest neighbor, caliper = 0.05, ratio = 3, replace = T, reuse.max=3 | 1678.14 | 3688 | 0.0279 | 1.0357 | 0 of 44 |
